# UBQLN4 promotes STING proteasomal degradation during cisplatin‐induced DNA damage in triple‐negative breast cancer

**DOI:** 10.1002/ctm2.985

**Published:** 2022-07-15

**Authors:** Yoshiaki Shoji, Takamichi Yokoe, Yuta Kobayashi, Tomohiro Murakami, Peter J. Bostick, Yosef Shiloh, Dave S. B. Hoon, Matias A. Bustos

**Affiliations:** ^1^ Department of Translational Molecular Medicine Saint John's Cancer Institute Santa Monica California USA; ^2^ Mayo Clinic Care Network Baton Rouge General Medical Center Baton Rouge Louisiana USA; ^3^ David and Inez Myers Laboratory for Cancer Genetics Tel Aviv University School of Medicine Tel Aviv Israel

To the Editor:

Cisplatin is a platinum agent that causes DNA damage and it is used as a single agent or in combination for the treatment of recurrent/unresectable triple‐negative breast cancer (TNBC).^1^ There is a renewed interest in cisplatin usage to treat TNBC in neoadjuvant/metastatic settings.^2^ Treatment options become limited when patients develop resistance, thus new insights into the molecular mechanisms driving cisplatin resistance will improve TNBC patient outcomes.

The aim of this study is to unravel novel molecular mechanisms controlling STING protein levels during cisplatin treatment. We *hypothesised* that during cisplatin‐induced DNA damage, STING is recognised by UBQLN4, and degraded through the ubiquitin‐proteasome system. *UBQLN4* mRNA expression was analysed in the TCGA BRCA and GTEx datasets. *UBQLN4* levels were significantly higher in primary TNBC tumours (Figure 1A,B). Patients with high *UBQLN4* mRNA levels had significantly reduced relapse‐free survival (RFS) (Figure 1C). *UBQLN4* gene is in the 1q22 region, and the amplification of the 1q arm is a frequent event in breast cancer (BC) and other solid tumours.^3^, ^4^ *UBQLN4* copy number variation (CNV) and mRNA levels showed a significant positive correlation in TNBC tumours and cell lines (Figure 1D,E). Immunohistochemistry analysis demonstrated significant elevated UBQLN4 protein levels for TNBC tumours (Figure 1F,G and Figure S1A). We have previously reported that UBQLN4 levels affect cisplatin sensitivity.^5^, ^6^ *UBQLN4* levels were associated with increased cisplatin resistance in TNBC cell lines (Figure 1H). *UBQLN4* depletion did not induce significant transcriptional changes in TNBC cell lines (Figure S1B–F) or reduce cellular proliferation (Figure S1G‐J); however, it increased the sensitivity to cisplatin, whereas *UBQLN4*‐OV led to cisplatin resistance (Figure 1I–K). In summary, the *UBQLN4* gene amplification correlates with increased UBQLN4 levels that led to cisplatin resistance in TNBC cell lines.

**FIGURE 1:**
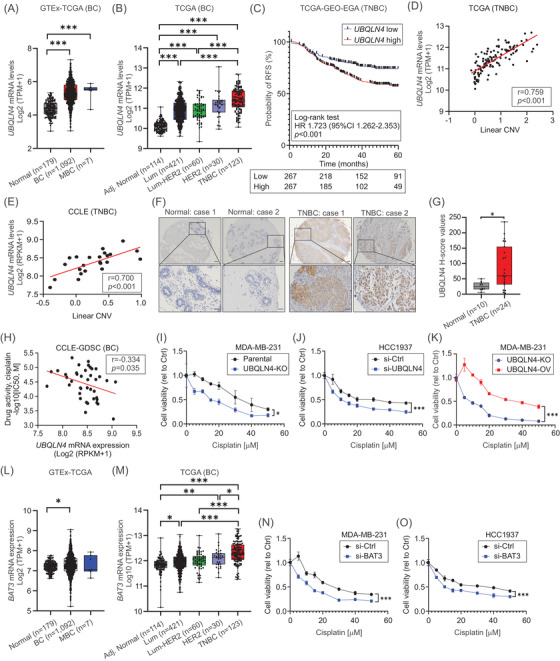
UBQLN4 levels determine cisplatin sensitivity in TNBC. (A) *UBQLN4* mRNA levels in normal breast (Normal), primary BC (BC), and metastatic BC (MBC) tissues in the TCGA and GTEx databases (one‐way ANOVA and Tukey's multiple comparisons test). (B) *UBQLN4* mRNA levels in tissues from the tumour‐adjacent normal breast (Normal), luminal (Lum), luminal‐HER2 (Lum‐HER2), HER2 and TNBC subtypes in the TCGA BRCA database (one‐way ANOVA and Tukey's multiple comparisons test). (C) Relapse‐Free Survival (RFS) analysis for TNBC patients with low (*n* = 267) versus high (*n* = 267) *UBQLN4* mRNA levels in TCGA, GEO and EGA databases combined. (D) Correlation between *UBQLN4* copy number variation (CNV) and *UBQLN4* mRNA levels for 119 TNBC tissues in the TCGA BRCA database. (E) Correlation between CNV and *UBQLN4* mRNA levels for 23 TNBC cell lines in the CCLE database. (F) (top) Representative images of UBQLN4 IHC for tumour‐adjacent normal breast (Normal) and primary TNBC tissues in the BC TMA. Scale bar = 50 μm. (down) Magnification of the representative images. Scale bar = 20 μm. (G) Quantification of *H*‐score values (Mann–Whitney *U* test). (H) Correlation between *UBQLN4* mRNA levels and cisplatin activity for 40 BC cell lines in the CCLE and GDSC databases. (I–K) Drug sensitivity assays comparing MDA‐MB‐231 parental and *UBQLN4*‐KO (I), HCC1937 si‐Ctrl and si‐UBQLN4 (J), and MDA‐MB‐231 *UBQLN4*‐KO and *UBQLN4*‐OV (K) cell lines treated with different cisplatin concentrations (two‐way ANOVA and Sidak's multiple comparisons test). (L) *BAT3* mRNA levels in normal breast (Normal), primary BC (BC) and metastatic BC (MBC) tissues in the TCGA and GTEx databases (one‐way ANOVA and Tukey's multiple comparisons test). (M) mRNA levels in tissues from the tumour‐adjacent normal breast (Normal), luminal (Lum), luminal‐HER2 (Lum‐HER2), HER2 and TNBC subtypes in the TCGA BRCA database (one‐way ANOVA and Tukey's multiple comparisons test). (N and O) Drug sensitivity assays comparing si‐Ctrl and si‐BAT3 in MDA‐MB‐231 (N) and HCC1937 (O) cell lines treated with different cisplatin concentrations (two‐way ANOVA and Sidak's multiple comparisons test). Drug sensitivity assays in each cell line were performed in replicates (*n* = 3)

UBQLN4 interacts with the chaperone protein BAT3 to reduce proteotoxic cell stress by translocating misassembled ER‐localised proteins for proteasomal degradation.^7^ *BAT3* mRNA levels were significantly higher in primary TNBC tumours (Figure 1L,M), but high *BAT3* mRNA levels did not associate with RFS (Figure S2A). *BAT3* knockdown did not affect UBQLN4 levels or cellular proliferation (Figure S2B–E), but increased the sensitivity to cisplatin (Figure 1N,O). Cisplatin treatment significantly increased γ‐H2AX foci‐formation in *UBQLN4*‐KO and *BAT3*‐knockdown, but not in *UBQLN4*‐OV cell lines (Figure S3A,B). Ubiquitination of BAT3‐captured proteins is required for efficient protein degradation.^8^ Under cisplatin treatment and proteasomal degradation blockage, BAT3 co‐immunoprecipitated with UBQLN4 in *UBQLN4*‐OV and parental cell lines (Figure S3C,D,F,G). Under the same conditions, UBQLN4 and BAT3 co‐immunoprecipitated with ubiquitinated DDK‐tagged proteins (Figure S3E). These results suggested that UBQLN4 and BAT3 interact with specific ubiquitinated proteins during cisplatin treatment in TNBC cell lines.

STING ubiquitination is required to initiate cytosolic DNA‐mediated activation.^9^, ^10^ Therefore, we hypothesised that in response to cisplatin‐induced DNA damage, STING is activated, ubiquitinated and regulated by UBQLN4‐mediated proteasomal degradation. *STING* mRNA levels were significantly lower in primary TNBC tumours (Figure 2A,B). Patients with low *STING* mRNA levels had significantly poor RFS (Figure 2C). Increased phosphorylated TBK1 levels were observed after cisplatin treatment, suggesting the STING pathway activation (Figure 2D). STING protein levels showed a significant positive correlation with cisplatin response (Figure 2E). *STING*‐knockdown did not affect UBQLN4/BAT3 levels or cellular proliferation (Figure S4A–C), but increased cisplatin resistance (Figure 2F,G). In conclusion, STING downregulation led to cisplatin resistance in TNBC cell lines.

**FIGURE 2:**
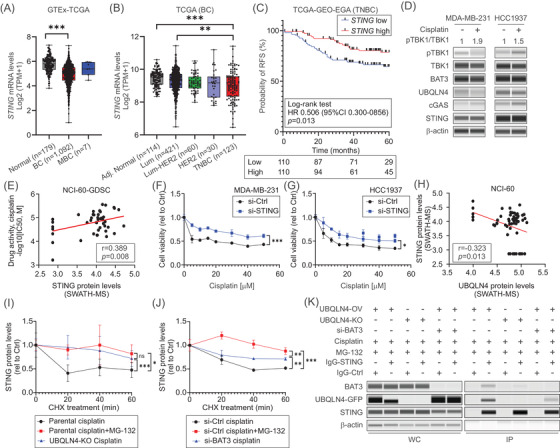
UBQLN4 promotes STING proteasomal degradation during cisplatin treatment. (A) *STING* mRNA levels in normal breast (Normal), primary BC (BC) and metastatic BC (MBC) tissues in the TCGA and GTEx databases (one‐way ANOVA and Tukey's multiple comparisons test). (B) *STING* mRNA levels in tissues from the tumour‐adjacent normal breast (Normal), luminal (Lum), luminal‐HER2 (Lum‐HER2), HER2 and TNBC subtypes in the TCGA BRCA database (one‐way ANOVA and Tukey's multiple comparisons test). (C) Relapse‐Free Survival (RFS) analysis for TNBC patients with low (*n* = 110) versus high (*n* = 110) *STING* mRNA expression in TCGA, GEO and EGA databases combined. (D) Western blotting analysis for STING pathway molecules (pTBK1, TKB1, BAT3, UBQLN4, cGAS and STING) and β‐actin (loading control) in TNBC cell lines untreated or treated with cisplatin (5 μM, 8 hours). pTBK1/TBK1 ratio was quantified relative to respective controls. (E) Correlation between STING protein levels and cisplatin activity for 45 cancer cell lines in the NCI‐60 and GDSC‐MGH‐Sanger datasets. Protein levels were determined using SWATH‐mass spectrophotometry. (F and G) Drug sensitivity assays comparing si‐Ctrl and si‐STING in MDA‐MB‐231 (F) and HCC1937 (G) cell lines treated with different cisplatin concentrations (two‐way ANOVA and Sidak's multiple comparisons test). (H) Correlation between UBQLN4 and STING protein levels for 59 cancer cell lines in the NCI‐60 dataset. (I and J) Quantification of STING protein levels for CHX assay in MDA‐MB‐231 *UBQLN4*‐KO (I), si‐BAT3 (J), and the respective control cell lines treated with cisplatin (5 μM) ± MG‐132 (5 μM) for 8 hours (two‐way ANOVA and Sidak's multiple comparisons test). (K) Co‐immunoprecipition (Co‐IP) assay in MDA‐MB‐231 *UBQLN4*‐OV, *UBQLN4*‐KO and *UBQLN4*‐OV+si‐BAT3 cell lines treated with cisplatin (5 μM) ± MG‐132 (5 μM) for 8 hours. Co‐IPs were performed using STING or control Ab. Protein levels were assessed in whole‐cell lysates (WC) and co‐IP fractions (IP). Drug sensitivity and CHX assays in each cell line were performed in replicates (*n* = 3)

UBQLN4 and STING protein levels showed a significant inverse correlation (Figure 2H). STING protein levels were decreased by cisplatin treatment and the blockage of the proteasomal degradation increased STING levels in *UBQLN4*‐OV, but not in *UBQLN4*‐KO cell lines (Figure S4D), suggesting that STING levels are controlled by UBQLN4‐mediated degradation. STING protein levels significantly increased by cisplatin treatment in *UBQLN4*‐KO and partially increased in *BAT3*‐knockdown cell lines compared to respective controls (Figure 2I,J and Figure S4E,F). Importantly, UBQLN4 co‐immunoprecipitated with STING during both the presence/absence of BAT3, suggesting a BAT3‐independent UBQLN4‐STING interaction (Figure 2K). G10 is a well‐established human‐specific STING agonistthat activated STING pathway in TNBC cell lines (Figure S4G).Also, UBQLN4 status determined G10 response (Figure 3A,B). During G10 treatment, UBQLN4/BAT3 interacted with STING (Figure 3C). Confocal microscopy was utilised to assess the co‐localisation of UBQLN4, STING and ubiquitinated proteins (DDK‐tagged). Increased STING ubiquitination, as well as significantly higher co‐localisation rates, were observed for UBQLN4‐ubiquitinated proteins (DDK‐tagged) UBQLN4‐STING, UBQLN4‐BAT3 and STING‐BAT3 during cisplatin or G10 treatment (Figure 3D,E). In summary, UBQLN4 mediates ubiquitinated STING proteasomal degradation during STING activation.

**FIGURE 3:**
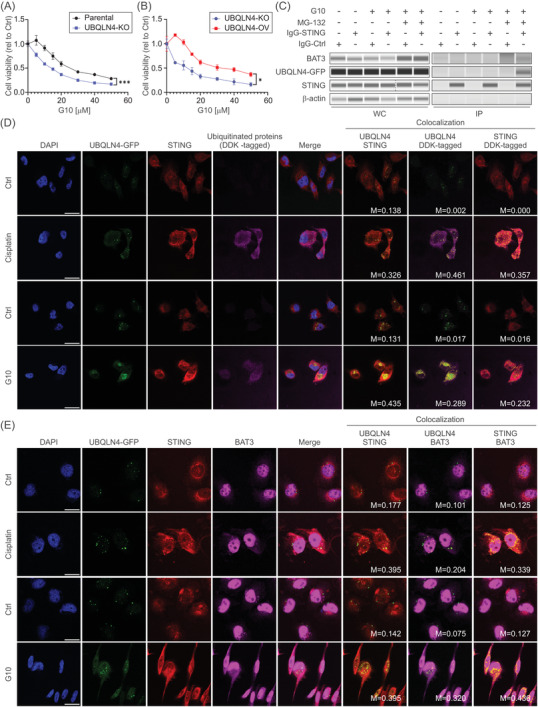
UBQLN4 interacts with ubiquitinated STING during STING activation. (A and B) Drug sensitivity assays comparing MDA‐MB‐231 parental and *UBQLN4*‐KO (A), *UBQLN4*‐KO and UBQLN4‐OV (B) cell lines treated with different G10 concentrations (two‐way ANOVA and Sidak's multiple comparisons test). (C) Co‐IP assay in MDA‐MB‐231 UBQLN4‐OV cell lines untreated or treated with G10 (25 μM) ± MG‐132 (5 μM) for 8 hours. Co‐IPs were performed using STING or control Ab. Protein levels were assessed in whole‐cell lysates (WC) and co‐IP fractions (IP). (D) Immunofluorescence staining for STING and DDK was performed in MDA‐MB‐231 UBQLN4‐OV cell lines untreated or treated with cisplatin (5 μM) or G10 (25 μM) plus MG‐132 (5 μM) for 8 hours. Representative images are shown for STING (Cy3, red), DDK (AF647, magenta), nucleus (DAPI, blue), UBQLN4 (GFP, green), and the merged images for each condition. Co‐localisation of UBQLN4‐STING, UBQLN4‐DDK and STING‐DDK are indicated in yellow. Manders’ overlap coefficients (M) are indicated in each image. Scale bar = 20 μm. (E) Immunofluorescence staining for STING and BAT3 performed in cisplatin (5 μM, 8 hours) or G10 (25 μM, 8 hours) treated or non‐treated MDA‐MB‐231 UBQLN4‐OV cell lines. Representative images are shown for STING (Cy3, red), BAT3 (AF647, magenta), nucleus (DAPI, blue), UBQLN4 (GFP, green), and the merged images for each condition. Co‐localisation of UBQLN4‐STING, UBQLN4‐BAT3 and STING‐BAT3 are indicated in yellow. Manders’ overlap coefficients (M) are indicated in each image. Scale bar = 20 μm. Drug sensitivity assays in each cell line were performed in replicates (*n* = 3)

Multiplex immunofluorescence for UBQLN4 and STING were performed on primary TNBC FFPE tissues (Table S1) and analysed by confocal microscopy (Figure S5A). A significant inverse correlation was observed between UBQLN4 and STING protein levels (Figure S5B). STING levels were significantly higher in TNBC tumours with low UBQLN4 (Figure S5C). We then assessed TNBC PDX models stratified into two treatment groups according to cisplatin responses (Tables S2 and S3 and Figure 4A). *UBQLN4* mRNA levels were significantly higher, whereas *STING* mRNA levels were significantly lower in tumour samples that had a non‐complete response (non‐CR, Figure 4B–D). As a readout of STING activation, IL‐6 levels were evaluated. In silico analysis showed that the *IL6* and *STING* mRNA levels positively correlated, while the *IL6* and *UBQLN4* mRNA levels negatively correlated (Figure S5D,E). Also, *UBQLN4*‐KO cell lines treated with cisplatin or G10 showed enhanced IL6 protein levels (Figure S5F,G).

**FIGURE 4:**
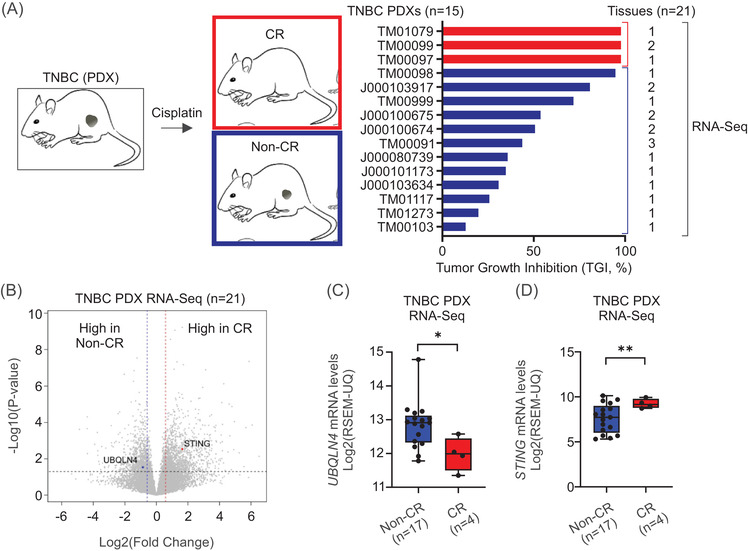
*UBQLN4* and *STING* mRNA levels predict cisplatin response in vivo. (A) RNA‐seq data were obtained from 21 samples across 15 primary TNBC PDX models, which were tested for cisplatin response. TNBC PDX models were treated by 2 mg/kg cisplatin (treated tumours, *n* = 8–11) or 5% dextrose in water, 5 ml/kg (control tumours, *n* = 6–11), 1/week, for three courses. Tumours with complete or partial responses were defined as responders, whereas stable diseases or progressive diseases were defined as non‐responders. (B) Volcano plot showing the transcriptomic changes in the group using the RNA‐seq data obtained from 21 TNBC PDX samples. *UBQLN4* and *STING* are indicated in blue and red, respectively. (C and D) Comparison of *UBQLN4* (C) and *STING* (D) mRNA levels in TNBC tissues from responders and non‐responders obtained from TNBC PDX mouse models (Student's *t*‐test)

In conclusion, *UBQLN4* locus amplification elevates UBQLN4 mRNA/protein levels that correlate with low STING mRNA/protein levels in TNBC tumours. Mechanistically, UBQLN4 delivers STING to proteasomal degradation during cisplatin or STING agonist treatment and promotes cisplatin resistance in vitro and in vivo (Figure S6). UBQLN4 is a novel factor regulating STING protein levels during STING pathway activation and may represent a predictive biomarker for cisplatin response in TNBC.

## CONFLICT OF INTEREST

The authors declare that there is no conflict of interest.

## Supporting information

Supporting Information
