# Supplementary material for: UBQLN4 promotes STING proteasomal degradation during cisplatin‐induced DNA damage in triple‐negative breast cancer
Source: Clin Transl Med. 2022 Jul 15;12(7):e985. doi: 10.1002/ctm2.985 (PMC9286529; doi:10.1002/ctm2.985)
Supplement: Supplementary file 1 — Supporting Information [file CTM2-12-e985-s001.docx]

**UBQLN4 Promotes STING Proteasomal Degradation During Cisplatin-Induced DNA Damage in Triple-Negative Breast Cancer**

Yoshiaki Shoji^1^, Takamichi Yokoe^1^, Yuta Kobayashi^1^, Tomohiro Murakami^1^, Peter J. Bostick^2^, Yosef Shiloh^3^, Dave S. B. Hoon^1^, and Matias A. Bustos^1*^

**Supplementary Information**

**Supplementary Material and Methods**

**Supplementary Tables**

**Table S1. Surgically Resected Primary TNBC Tumors Analyzed by Multiplex IF**

**Table S2. TNBC PDX Mouse Models**

**Table S3. Reagents and Resources**

**Supplementary Figures**

**Figure S1. UBQLN4 is upregulated in TNBC**

**Figure S2. UBQLN4 binds to BAT3 and ubiquitinated proteins during cisplatin-induced DNA damage**

**Figure S3. UBQLN4 and BAT3 interact during cisplatin treatment.**

**Figure S4. UBQLN4 mediates STING proteasomal degradation**

**Figure S5. UBQLN4 regulates STING levels and downstream activation**

**Figure S6. Graphical abstract**

**Figure S7. Uncropped western blotting images**

**Figure S8. Uncropped western blotting images**

**Figure S9. Uncropped western blotting images**

**Figure S10. Uncropped western blotting images**

**Figure S11. Uncropped western blotting images**

**Supplementary Material and Methods**

**Ethics approval and consent to participate**

All the studies followed the World Medical Association Declaration of Helsinki and the NIH Belmont Report principles. All human samples and clinical information for this study were obtained according to the IRB protocol guidelines approved by Saint John’s Health Center (SJHC) Joint Institutional Review Board (IRB) and Western IRB: MORD-RTPCR-0995. All specimens in the study were de-identified and HIPAA regulations were followed.

**Breast cancer tissues and cell lines**

A clinically annotated tissue microarray (TMA, BC081120c) for BC and adjacent normal breast tissues was obtained from US Biomax (Derwood, MD). Formalin-fixed paraffin-embedded (FFPE) tissues from 10 surgically resected TNBC primary tumors were obtained from patients who underwent up-front surgery at Baton Rouge General Medical Center, Baton Rouge, LA (**Tables S1 and S3**).

Established human BC cell lines (MDA-MB-231 and HCC1937) were obtained from the American Type Culture Collection (ATCC, Manassas, VA) and were cultured as recommended. All human cell lines have been authenticated using short tandem repeat profiling within the last three years. All experiments were performed with mycoplasma-free cell lines.

**Mice and TNBC patient-derived xenograft**

Mice experiments were performed by The Jackson Laboratory (Bar Harbor, ME) following the Guide for the Care and Use of Laboratory Animals. Detailed information for the patient-derived xenograft (PDX) models is available at the Mouse Models of Human Cancer Database ^1^. The Upper quantile normalized RNA-seq by expectation-maximization (RSEM) RNA-seq data was provided from The Jackson Laboratory. Treatment response for cisplatin and the details of the primary TNBC tumors were available in 15 PDX models by the Mouse Models of Human Cancer Database ^1^. TNBC PDX models were treated by 2 mg/kg cisplatin (treated tumors, n=8-11) or 5% dextrose, 5 ml/kg (control tumors, n=6-11), 1/week, for 3 courses. Tumor growth inhibition (TGI) and treatment responses for each model were defined as previously described ^2^. Models with complete response (CR) were classified as CR, whereas partial response (PR), stable disease (SD), or progressive disease (PD) were classified as non-CR. RNA-seq data for 21 samples across 15 TNBC PDX mouse models were analyzed.

**CRISPR UBQLN4 knockouts**

MDA-MB-231 cells were tested for sensitivity to blasticidin S (Thermo Fisher Scientific, Waltham, MA and InvivoGen, San Diego, CA) in dose-response curves. Cell lines were transduced with lentivirus particles (Horizon Discovery, Waterbeach, UK) to overexpress Cas9. Cas9-positive cell lines were selected for 10 days in a medium containing blasticidin S.

Specific CRISPR RNA (crRNA) were designed to target exon 1 and exon 6 of the *UBQLN4* gene using the MIT web tool (<http://crispr.mit.edu/>). The combination of crRNA (Horizon Discovery) and trans-activating crRNA (tracrRNA, Horizon Discovery) was used for CRISPR guide selection. Thirty to fifty thousand MDA-MB-231 Cas9-positive cells were plated and transfected with 50 nM of each crRNA/tracrRNA according to the manufacturer’s instructions.

**RNA isolation, RT-qPCR, and RNA-seq**

RNA was extracted by the ZR-Duet™ DNA/RNA MiniPrep kit (Zymo Research, Irvine, CA) according to the manufacturer’s instructions. The details of reverse transcription quantitative polymerase chain reaction (RT-qPCR) have been previously described ^3^. Primer sets used in RT-qPCR are shown in **Table S3**.

RNA sequencing (RNA-seq) was performed in triplicates for MDA-MB-231 parental and UBQLN4-knockout (KO) cell lines as previously described ^3,4^. The quality of RNA samples was estimated by RNA integrity number using Agilent 2200 TapeStation (Agilent Technologies, Santa Clara, CA). High-quality RNA (RNA integrity number > 8.0) was used to create mRNA libraries using the Illumina TruSeq RNA Sample Preparation Kit v2 (San Diego, CA). The libraries were sequenced on the Illumina HiSeq 2500 (Illumina Inc.) in Rapid Mode using 50 bp single-end reads and achieved an average read depth of over 30 million reads per sample at the SJCI Sequencing Center. Sequencing Reads were mapped to the GENCODE Release 19 (<https://www.gencodegenes.org/human/release_19.html>) using STAR version 2.6.1c (https://github.com/alexdobin/STAR), and read counts were generated using the quantMode GeneCounts option in STAR. The expression levels of each gene were calculated using DESeq2 (<http://bioconductor.org/packages/release/bioc/html/DESeq2.html>) and differentially expressed genes were screened using log2 foldchange (log2FC) >1 or <-1.

**Plasmids**

*Ubiquitin B* (UBB) pCMV6 vector and the empty vector were from OriGene, Rockville, MD. *UBQLN4* was cloned in pLNCX2 plasmid as described previously ^5^. *UBQLN4* or empty vectors were packaged into lentivirus particles using HEK-293T (ATCC) cell lines to obtain stable clones. Purified lentiviral particles were transduced into MDA-MB-231 *UBQLN4*-KO cell lines. Stable clones were selected with G418 (Thermo Fisher Scientific).

Knockdown experiments were performed as previously described ^6^. HCC1937 cell lines were transfected with 20 nM pool siRNA targeting *UBQLN4* or non-targeting control (Horizon Discovery) using jetPRIME transfection reagent (Polypus-transfection, Illkirch, France). MDA-MB-231 and HCC1937 cell lines were transfected with 50 nM pool siRNA targeting BAT3 or non-targeting control (Horizon Discovery) using jetPRIME transfection reagent. MDA-MB-231 and HCC1937 cell lines were also transfected with 50 nM pool siRNA targeting STING or non-targeting control (Horizon Discovery) using jetPRIME transfection reagent. Specific small inhibitory RNA (siRNA) used in each experiment are shown in **Table S3**.

**Drug treatment**

Cisplatin (Selleck Chemicals, Houston, TX) was dissolved in water at a concentration of 4 mM. Human-specific STING agonist 4-[(2-Chloro-6-fluorophenyl) methyl]-N-(2-furanylmethyl)-3,4-dihydro-3-oxo-2H-1,4-benzothiazine-6-carboxamide (G10, Bio-Techne, Minneapolis, MN) was dissolved in 0.05% dimethyl sulfoxide (DMSO) at a concentration of 10 mM. Proteasome inhibitor MG-132 (Selleck Chemicals) was dissolved in water at a concentration of 10 mM. For cell viability assays, the measurements were performed before and after treatment with different concentrations (0, 5, 10, 15, 20, 30, 40, and 50 µM) of cisplatin and G10 for 24 hours.

**Immunohistochemistry (IHC)**

BC TMA was stained with UBQLN4 antibody (Ab) as previously described ^7^. Images were taken using a Revolve microscope and the ECHO Pro App (ECHO Laboratories, San Diego, CA). H-scores were calculated using QuPath v.0.3.0 (Queen's University, Belfast, Northern Ireland). Cell detection and quantification were conducted using QuPaths built-in “Positive cell detection” ^8^.

FFPE slides from 10 surgically resected TNBC primary tumors were obtained from patients who underwent up-front surgery at Baton Rouge General Medical Center, Baton Rouge, LA (**Tables S1 and S3**) were stained for multiplex IF. Deparaffinization, rehydration, and antigen retrieval were performed as previously described ^6,7^. Slides were blocked using Buffer W (NanoString Technologies, Seattle, WA), and then stained with fluorescence conjugated UBQLN4, STING, and pan cytokeratins (panCK) Ab. Ab dilutions are shown in **Table S3.** Slides were mounted with Mowiol 4-88 mounting media containing 2 µg/ml 4’, 6’-diaminodino-2-phenylindole (DAPI, Thermo Fisher Scientific). Confocal images were acquired on Leica TCS-SP8 inverted spectral confocal microscope (Leica Microsystems, Wetzlar, Germany). Images were processed using Leica Application Suite X (Leica Microsystems) and merged using Fiji software ^9^. Cell detection and quantification were conducted using QuPaths built-in “Cell detection” ^8^.

**Co-immunoprecipitation (co-IP) assays**

Co-IP assays were performed as previously described ^6^. Cell lines were transfected with UBB plasmids as indicated in each experiment and were treated with 5 μM cisplatin/25 μM G10 ± 5 μM MG-132 for 8 hours. After drug treatment, cell lines were washed and lysed in co-IP buffer (150 mM NaCl, 100 mM Tris-HCl pH 8, 1% NP-40, protease, and phosphatase inhibitors) by gently pipetting. The whole-cell lysates were quantified by bicinchoninic acid assay, and 250 μg of the whole-cell lysate (final concentration 1.25 μg/μL) were incubated with UBQLN4 and mouse (G3A1) control Ab, or BAT3 and mouse (G3A1) control Ab, or DDDDK tag and normal rabbit control Ab, or STING and normal rabbit control Ab in the co-IP buffer. Immune complexes were further incubated with Dynabeads™ Protein G (Thermo Fisher Scientific) overnight at 4 ºC. The beads were washed 3X with co-IP buffer, recovered, and boiled in the fluorescent master mix for 5 min at 95ºin a dry bath. After centrifugation, the supernatants were recovered, and protein concentration was adjusted to 1 μg/μL with a 1x fluorescent master mix reagent. Samples were analyzed by an automated western blotting system as described below. Ab dilutions are shown in **Table S3**.

**Immunofluorescence (IF) on cell lines**

Cell lines were transfected with UBB plasmids as indicated in each experiment. TNBC cell lines (1 x 10^4^) were seeded in 8-well Falcon™ chambered culture slides (Thermo Fisher Scientific), followed by 5 μM cisplatin/25 μM G10 ± 5 μM MG-132 treatment for 8 hours. The protocol was performed as previously described ^6^. Ab dilutions are shown in **Table S3**. Confocal images were taken and processed as described above. Fluorescence images for gamma-H2A.X variant histone (γ-H2AX) were taken on a Revolve microscope. Fluorescence intensity for each cell was quantified, and all images were processed using the Fiji software. Colocalization of the particles for UBQLN4-STING, UBQLN4-DDK, and STING-DDK or UBQLN4-STING, UBQLN4-BAT3, and STING-BAT3 was analyzed using the Fiji plugin JACoP ^10^.

**Cell viability assay**

TNBC cell lines (1 x 10^3^) were seeded in a 96-well plate. The number of viable cells was assessed using a Cell Titer-Glo Luminescent (Promega, Madison, WI) as previously described ^3,6^ according to the manufacturer’s instructions.

**Cycloheximide chase (CHX) assays**

Cells were incubated with 100 μg/mL cycloheximide (Millipore Sigma, Darmstadt, Germany) and 5 μM cisplatin ± 5 μM MG-132 at different time points (0, 20, 40, and 60 minutes). Cells were harvested and processed for protein extraction. STING protein levels were quantified over time by western blotting as described below.

**Western blotting**

Automated western blotting was performed according to the manufacturer’s protocol (Protein Simple, San Jose, CA), and quantified as previously described ^6,11^. The proteins were extracted with lysis buffer (150 mM NaCl, 100 mM Tris-HCl pH 8, 1% NP-40, phosphatase, and protease inhibitors) and the protein concentration was adjusted to 0.5 μg/μL. The results were analyzed using the Compass Software (Protein Simple) with β-actin levels serving as the loading control. Ab dilutions are shown in **Table S1**. All uncropped western blotting images are shown in **Figure S7-S11**.

**Enzyme-linked immunoassay (ELISA)**

TNBC cell lines (2 x 10^5^) were seeded in a 6-well plate and were incubated for 24 hours. Cells were either treated by 5 μM cisplatin, 25 μM G10, or DMSO for 12 hours. Interleukin-6 (IL-6) levels in the supernatant were quantified using the IL-6 Human ELISA Kit (Thermo Fisher Scientific) according to the manufacturer’s instructions.

**Analysis of public datasets**

Datasets from The Cancer Genome Atlas (TCGA) breast cancer (BRCA) ^12^ and Genotype-Tissue Expression (GTEx) Breast-Mammary Tissue ^13^ were downloaded through the University of California Santa Cruz (UCSC) Xena ^14^. Datasets from The Cancer Cell Line Encyclopedia (CCLE) ^15^, Genomics of Drug Sensitivity in Cancer (GDSC) ^16^, and National Cancer Institute (NCI)-60 Human Tumor Cell Lines Screen was downloaded through CellMiner Cross-Database (CellMinerCDB) Version 1.4 ^17^. Survival analyses were performed with the Kaplan-Meier Plotter ^18^.

**Biostatistics and bioinformatics analysis**

Statistical analyses were performed using the GraphPad Prism 8 (GraphPad software, San Diego, CA) or R version 4.1.2 (https://www.R-project.org/.) in a two-tailed way. The distribution and variation within each group of data were assessed before statistical analysis. Two groups were compared using Student’s *t*-test or the Mann-Whitney U test. Multiple groups were analyzed by One-way or Two-way ANOVA followed by post-hoc tests. The correlation was determined using Pearson’s correlation test. Colocalization analysis was performed using Manders' overlap coefficient ^10^. Recurrence-free survival (RFS) was calculated from the time of taking the first specimen until recurrence or last contact with the patient and analyzed using the log-rank test. Patients were divided into two groups using the median expression for each mRNA. The volcano plot was used for the RNA-seq data to demonstrate the global transcriptional change between groups. A two-sided *p*<0.05 was considered statistically significant: *=*p<*0.05, **=*p<*0.01, ***=*p<*0.001, and ns=not significant. All figures were unified using Adobe Illustrator CC (Adobe, San Jose, CA) or CorelDraw graphics suite 8X (Corel, Ottawa, Canada).

**List of abbreviations**

Ab: antibody, AJCC: American Joint Committee on Cancer, ANOVA: analysis of variance, ATCC: American Type Culture Collection, BAT3: HLA-B associated transcript 3, BC: breast cancer, β-actin: beta actin, CCLE: Cancer Cell Line Encyclopedia, CellMinerCDB: CellMiner Cross-Database, CMV: cytomegalovirus, CNV: copy number variation, co-IP: co-immunoprecipitation, CRISPR: clustered regularly interspaced short palindromic repeat, crRNA: CRISPR-RNA, DAB: 3,3’-diaminobenzidine, DAPI: 4’,6-diamidino-2-phenylindole, DDK: Dbf4-dependent protein kinase, DMSO: dimethyl sulfoxide, EGA: European Genome-Phenome Archive, ELISA: enzyme-linked immunoassay, ER: endoplasmic reticulum, ESCC: esophageal squamous cell carcinoma, FFPE: formalin-fixed paraffin-embedded, GDSC: Genomics of Drug Sensitivity in Cancer, GEO: Gene Omnibus Expression, GTEx: Genotype-Tissue Expression, G10: 4-[(2-Chloro-6-fluorophenyl)methyl]-N-(2-furanylmethyl)-3,4-dihydro-3-oxo-2H-1,4-benzothiazine-6-carboxamide, HER2: human epidermal growth factor receptor 2, HR: hazard ratio, IDC: invasive ductal carcinoma, IF: immunofluorescence, IHC: immunohistochemistry, IL: interleukin, KO: knockout, LVI: lymphovascular invasion, MBC: metastatic breast cancer, mRNA: messenger RNA, NCI: National Cancer Institute, OV: overexpression, panCK: pan cytokeratins, RFS: recurrence-free survival, RNA-seq: RNA sequencing, RSEM: RNA-seq by expectation-maximization, RT-qPCR: reverse transcription quantitative polymerase chain reaction, SDHA: succinate dehydrogenase complex flavoprotein subunit A, STING: stimulator of interferon genes, TBK1: TANK binding kinase 1, TCGA: The Cancer Genome Atlas, TMA: tissue microarray, TNBC: triple-negative breast cancer, tracrRNA: trans-activating crRNA, UBB: ubiquitin B, UBQLN4: Ubiquilin-4.

**Supplementary Tables**

**Table S1**. Surgically Resected Primary TNBC Tumors Analyzed by Multiplex IF

| **Case** | **Sex** | **Age** | **Laterality** | **Procedure** | **Tumor Size (cm)** | **Histological Type** | **Histological Grade** | **Nuclear Grade** | **LVI** | **pT** | **pN** | **ER** | **PR** | **HER2 Final** |
| --- | --- | --- | --- | --- | --- | --- | --- | --- | --- | --- | --- | --- | --- | --- |
| 1 | Female | 59 | Left | Lumpectomy | 2.1 | IDC | 9 | 3 | Suspicious | pT2 | pNX | 0 | 0 | Negative |
| 2 | Female | 45 | Left | Lumpectomy | 1.3 | IDC | 9 | 3 | Absent | pT1c | pN1a | 0 | 0 | Negative |
| 3 | Female | 46 | Left | Lumpectomy | 0.7 | IDC | 8 | 3 | Absent | ypT1b | ypN2a | 0 | 0 | Negative |
| 4 | Female | 50 | Right | Mastectomy | 1.9 | IDC | 9 | 3 | NA | pT1c | pN0 | 0 | 0 | Negative |
| 5 | Female | 59 | Right | Mastectomy | 3.1 | IDC | 9 | 3 | NA | pT2 | pN0 | 0 | 0 | Negative |
| 6 | Female | 74 | Left | Mastectomy | 1.7 | IDC | 7 | 2 | Present | pT1c | pN1mi | 0 | 0 | Negative |
| 7 | Female | 37 | Left | Lumpectomy | 1.1 | IDC | 9 | 3 | Absent | pT1c | pNX | 0 | 0 | Negative |
| 8 | Female | 46 | Left | Mastectomy | NA | IDC | NA | NA | NA | NA | pN1a | 0 | 0 | Negative |
| 9 | Female | 52 | Right | Lumpectomy | 1.3 | IDC | 9 | 3 | Absent | pTX | pNX | 0 | 0 | Negative |
| 10 | Female | 61 | Right | Lumpectomy | NA | IDC | NA | NA | NA | ypTX | ypN1mi | 0 | 0 | Negative |

IDC: invasive ductal carcinoma, LVI: lymphovascular invasion, NA: not available.

**Table S2**. TNBC PDX Mouse Models

| **Model** | **Model name** | **Sex** | **Age** | **Diagnosis** | **AJCC Stage** | **Grade** | **Previous Treatment** | **Sample Type** | **Host Strain** | **TGI (%)** | **Group** |
| --- | --- | --- | --- | --- | --- | --- | --- | --- | --- | --- | --- |
| 1 | J000080739 | Female | 49 | IDC | IV | 3 | Yes | Surgical resection | NOD.Cg-*Prkdc^scid^ Il2rg^tm1Wjl^*/SzJ | 36 | Non-CR |
| 2 | J000100674 | Female | 61 | IDC | IIIC | 3 | Yes | Surgical resection | NOD.Cg-*Prkdc^scid^ Il2rg^tm1Wjl^*/SzJ | 51 | Non-CR |
| 3 | J000100675 | Female | 56 | IDC | IIB | 3 | No | Surgical resection | NOD.Cg-*Prkdc^scid^ Il2rg^tm1Wjl^*/SzJ | 54 | Non-CR |
| 4 | J000101173 | Female | 55 | IDC | II | 3 | Yes | Biopsy | NOD.Cg-*Prkdc^scid^ Il2rg^tm1Wjl^*/SzJ | 35 | Non-CR |
| 5 | J000103634 | Female | 55 | IDC | II | 3 | Yes | Surgical resection | NOD.Cg-*Prkdc^scid^ Il2rg^tm1Wjl^*/SzJ | 31 | Non-CR |
| 6 | J000103917 | Female | 66 | IDC | IA | 3 | Yes | Surgical resection | NOD.Cg-*Prkdc^scid^ Il2rg^tm1Wjl^*/SzJ | 81 | Non-CR |
| 7 | TM00091 | Female | 45 | IDC | IIB | 3 | Yes | Surgical resection | NOD.Cg-*Prkdc^scid^ Il2rg^tm1Wjl^*/SzJ | 44 | Non-CR |
| 8 | TM00097 | Female | 60 | IDC | IIIA | 3 | No | Surgical resection | NOD.Cg-*Prkdc^scid^ Il2rg^tm1Wjl^*/SzJ | 98 | CR |
| 9 | TM00098 | Female | 64 | IDC | IA | 3 | No | Surgical resection | NOD.Cg-*Prkdc^scid^ Il2rg^tm1Wjl^*/SzJ | 95 | Non-CR |
| 10 | TM00099 | Female | 44 | IDC | IIA | 3 | Yes | Surgical resection | NOD.Cg-*Prkdc^scid^ Il2rg^tm1Wjl^*/SzJ | 98 | CR |
| 11 | TM00103 | Female | 73 | IDC | I | 3 | Yes | Biopsy | NOD.Cg-*Prkdc^scid^ Il2rg^tm1Wjl^*/SzJ | 13 | Non-CR |
| 12 | TM00999 | Female | 45 | IDC | I | 3 | No | Surgical resection | NOD.Cg-*Prkdc^scid^ Il2rg^tm1Wjl^*/SzJ | 72 | Non-CR |
| 13 | TM01079 | Female | 32 | IDC | IIB | 3 | No | Surgical resection | NOD.Cg-*Prkdc^scid^ Il2rg^tm1Wjl^*/SzJ | 98 | CR |
| 14 | TM01117 | Female | 51 | IDC | IIA | 3 | No | Surgical resection | NOD.Cg-*Prkdc^scid^ Il2rg^tm1Wjl^*/SzJ | 26 | Non-CR |
| 15 | TM01273 | Female | 65 | IDC | IIA | 3 | No | Surgical resection | NOD.Cg-*Prkdc^scid^ Il2rg^tm1Wjl^*/SzJ | 20 | Non-CR |

AJCC: American Joint Committee on Cancer, TGI: tumor growth inhibition, IDC: invasive ductal carcinoma

**Table S3**. Reagents and Resources

| **Reagent or Resource** | **Source** | **Catalog** | **Dilution** | | | | |
| --- | --- | --- | --- | --- | --- | --- | --- |
| **Antibodies** |  |  | **WES** | **IF** | **IP** | **IHC** | **M-IF** |
| Mouse monoclonal anti-β-actin | Millipore Sigma | A5441 | 1:500 |  |  |  |  |
| Mouse monoclonal anti-UBQLN4 | Santa Cruz Biotechnology | sc-136145 | 1:10 |  | 5 ug | 1:200 |  |
| Rabbit monoclonal anti-UBQLN4 mAb | BETHYL Laboratories | A700-145 | 1:10 |  |  |  |  |
| Rabbit monoclonal anti-UBQLN4 mAb | BETHYL Laboratories | A700-145D4 |  |  |  |  | 1:10 |
| Rabbit monoclonal anti-BAT3/BAG6 | Abcam | ab137076 | 1:400 |  |  |  |  |
| Mouse polyclonal anti-BAT3/BAG6 | Abcam | ab88292 | 1:400 | 1:100 | 5 ug |  |  |
| Rabbit monoclonal anti-cGAS (D1D3G) | Cell Signaling Technology | 15102 | 1:50 |  |  |  |  |
| Rabbit monoclonal anti-STING (D2P2F) | Cell Signaling Technology | 13647 | 1:50 |  |  |  |  |
| Rabbit monoclonal anti-STING | Abcam | ab239074 |  |  | 5 ug |  |  |
| Rabbit polyclonal anti-STING | Proteintech | 19851-1-AP |  | 1:100 |  |  |  |
| Rabbit polyclonal anti-STING | Proteintech | CL488-19851 |  |  |  |  | 1:100 |
| Rabbit polyclonal anti-DDDDK tag | Abcam | ab1162 | 1:50 |  | 5 ug |  |  |
| Mouse monoclonal anti-DDK (FLAG) | Origene | TA50011 |  | 1:100 |  |  |  |
| Rabbit monoclonal anti-TBK1/NAK (D1B4) | Cell Signaling Technology | 3504 | 1:50 |  |  |  |  |
| Rabbit monoclonal anti-TBK1/NAK (Ser172) (D52C2) XP® | Cell Signaling Technology | 5483 | 1:50 |  |  |  |  |
| Mouse monoclonal anti-pan cytokeratin (AE-1/AE-3) | Bio-Techne | NBP2-33200AF647 |  |  |  |  | 1:100 |
| Mouse monoclonal anti-phospho-histone H2A.X (Ser139) | Millipore Sigma | 05-636 |  | 1:100 |  |  |  |
| Goat Anti-Mouse Secondary HRP Conjugate | Protein Simple | 042-205 | 1 |  |  |  |  |
| Goat Anti-Rabbit Secondary HRP Conjugate | Protein Simple | 042-206 | 1 |  |  |  |  |
| Goat anti-Rabbit IgG (H+L), Cy™3 AffiniPure | Jackson ImmunoResearch Laboratories | 111-165-003 |  | 1:600 |  |  |  |
| Goat anti-Mouse IgG1 cross-absorbed, Alexa Fluor 647 | Thermo Fisher Scientific | A-21240 |  | 1:600 |  |  |  |
| Mouse (G3A1) monoclonal IgG1 isotype control | Cell Signaling Technology | 5415 |  |  | 5 ug |  |  |
| Rabbit IgG normal | Cell Signaling Technology | 2729 |  |  | 5 ug |  |  |
| **Chemicals, Peptides, and Recombinant Proteins** | | |  |  |  |  |  |
| Cisplatin | Selleck Chemicals | S1166 |  |  |  |  |  |
| MG-132 | Selleck Chemicals | S2619 |  |  |  |  |  |
| G10 | Bio-Techne | 5944 |  |  |  |  |  |
| Puromycin dihydrochloride | Thermo Fisher Scientific | A1113803 |  |  |  |  |  |
| Blasticidin S | Thermo Fisher Scientific | BP2647-25/ant-bl-1 |  |  |  |  |  |
| G418 | Thermo Fisher Scientific | 10131035 |  |  |  |  |  |
| DAPI | Thermo Fisher Scientific | 62248 |  |  |  |  |  |
| Buffer W | NanoString Technologies | N/A |  |  |  |  |  |
| Dynabeads™ Protein G | Thermo Fisher Scientific | 10003D |  |  |  |  |  |
| Cycloheximide | Millipore Sigma | C7698 |  |  |  |  |  |
| **Biological Samples** | | |  |  |  |  |  |
| TNBC FFPE tissues | This paper | Baton Rouge General Medical Center |  |  |  |  |  |
| BC TMA | US Biomax | BC081120c |  |  |  |  |  |
| **Experimental Models: Cell Lines** | | |  |  |  |  |  |
| Human MDA-MB-231 cell line | ATCC | HTB-26 |  |  |  |  |  |
| Human HCC1937 cell line | ATCC | CRL-2336 |  |  |  |  |  |
| **Experimental Models: Organisms/Strains** | | |  |  |  |  |  |
| Mouse: TNBC PDX | Jackson Laboratories | J000080739 |  |  |  |  |  |
| Mouse: TNBC PDX | Jackson Laboratories | J000100674 |  |  |  |  |  |
| Mouse: TNBC PDX | Jackson Laboratories | J000100675 |  |  |  |  |  |
| Mouse: TNBC PDX | Jackson Laboratories | J000101173 |  |  |  |  |  |
| Mouse: TNBC PDX | Jackson Laboratories | J000103634 |  |  |  |  |  |
| Mouse: TNBC PDX | Jackson Laboratories | J000103917 |  |  |  |  |  |
| Mouse: TNBC PDX | Jackson Laboratories | TM00091 |  |  |  |  |  |
| Mouse: TNBC PDX | Jackson Laboratories | TM00097 |  |  |  |  |  |
| Mouse: TNBC PDX | Jackson Laboratories | TM00098 |  |  |  |  |  |
| Mouse: TNBC PDX | Jackson Laboratories | TM00099 |  |  |  |  |  |
| Mouse: TNBC PDX | Jackson Laboratories | TM00103 |  |  |  |  |  |
| Mouse: TNBC PDX | Jackson Laboratories | TM00999 |  |  |  |  |  |
| Mouse: TNBC PDX | Jackson Laboratories | TM01079 |  |  |  |  |  |
| Mouse: TNBC PDX | Jackson Laboratories | TM01117 |  |  |  |  |  |
| Mouse: TNBC PDX | Jackson Laboratories | TM01273 |  |  |  |  |  |
| **Critical Commercial Assays** | | |  |  |  |  |  |
| CellTiter-Glo^®^ Luminescent Cell Viability Assay | Promega | G7572 |  |  |  |  |  |
| jetPRIME^TM^ transfection reagent | Polypus-transfection | 89129-924 |  |  |  |  |  |
| Pierce^TM^ BCA Protein Assay Kit | Life Technologies | 23227 |  |  |  |  |  |
| ZR-Duet™ DNA/RNA MiniPrep kit | Zymo Research | D7001 |  |  |  |  |  |
| Agilent 2200 TapeStation | Agilent Technologies | N/A |  |  |  |  |  |
| TruSeq RNA Sample Preparation Kit v2 | Illumina | RS-122-2001 |  |  |  |  |  |
| IL-6 Human ELISA Kit | Thermo Fisher Scientific | EH2IL6 |  |  |  |  |  |
| **Bacterial and virus strains** | | |  |  |  |  |  |
| Dharmacon™ Edit-R™ lentiviral Cas9 nuclease expression particles | Horizon Discovery | VCAS10124 |  |  |  |  |  |
| **Oligonucleotides** | | |  |  |  |  |  |
| ON-TARGETplus Human UBQLN4 siRNA | Horizon Discovery | L-021178-01-0005 |  |  |  |  |  |
| ON-TARGETplus Human BAT3 siRNA | Horizon Discovery | L-009271-00-0005 |  |  |  |  |  |
| ON-TARGETplus Human STING siRNA | Horizon Discovery | L-024333-00-0005 |  |  |  |  |  |
| ON-TARGETplus Non-targeting pool siRNA | Horizon Discovery | D-001810-10-05 |  |  |  |  |  |
| crRNA exon 1 | Horizon Discovery | CR-021178-01-0002 |  |  |  |  |  |
| crRNA exon 6 | Horizon Discovery | CR-021178-03-0002 |  |  |  |  |  |
| tracrRNA | Horizon Discovery | U-002005-20 |  |  |  |  |  |
| UBB (Myc-DDK-tagged)-Human ubiquitin B (UBB) | Origene | RC201747 |  |  |  |  |  |
| pCMV6-Entry, mammalian vector with C-terminal Myc- DDK Tag | Origene | PS100001 |  |  |  |  |  |
| **Software and Algorithms** | | |  |  |  |  |  |
| MIT web tool | ZHANG LAB | http://crispr.mit.edu/ |  |  |  |  |  |
| STAR aligner | N/A | v.2.6.1c |  |  |  |  |  |
| DESeq2 | N/A | DOI: 10.18129/B9.bioc.DESeq2 |  |  |  |  |  |
| Fiji | NIH | https://imagej.net/software/fiji/ |  |  |  |  |  |
| GraphPad Prism 8 | GraphPad Software | www.graphpad.com/ |  |  |  |  |  |
| R version 4.1.2 | The R Project | https://www.R-project.org/ |  |  |  |  |  |
| Adobe Illustrator CC | Adobe | www.adobe.com/ |  |  |  |  |  |
| CorelDraw graphics suite 8X | Corel | https://www.coreldraw.com/en/ |  |  |  |  |  |
| ECHO Pro App | ECHO Laboratories | https://discover-echo.com/ |  |  |  |  |  |
| QuPath v.0.3.0 | Queen's University | https://github.com/qupath |  |  |  |  |  |
| Leica Application Suite X | Leica Microsystems | https://www.leica-microsystems.com/ |  |  |  |  |  |
| Compass | Protein Simple | https://www.proteinsimple.com/ |  |  |  |  |  |
| **Primer sequence (5’-3’) for qPCR** | **Forward** | **Reverse** |  |  |  |  |  |
| *UBQLN4* | CAGATGCTGGCAGTGGAA | CCAAAGCCAGAGAGTATGGAC |  |  |  |  |  |
| *SDHA* | TCAGCATGCAGAAGTCAAT | GAACG TCTTCAGGTGCTTT |  |  |  |  |  |

**Supplementary Figures**

**
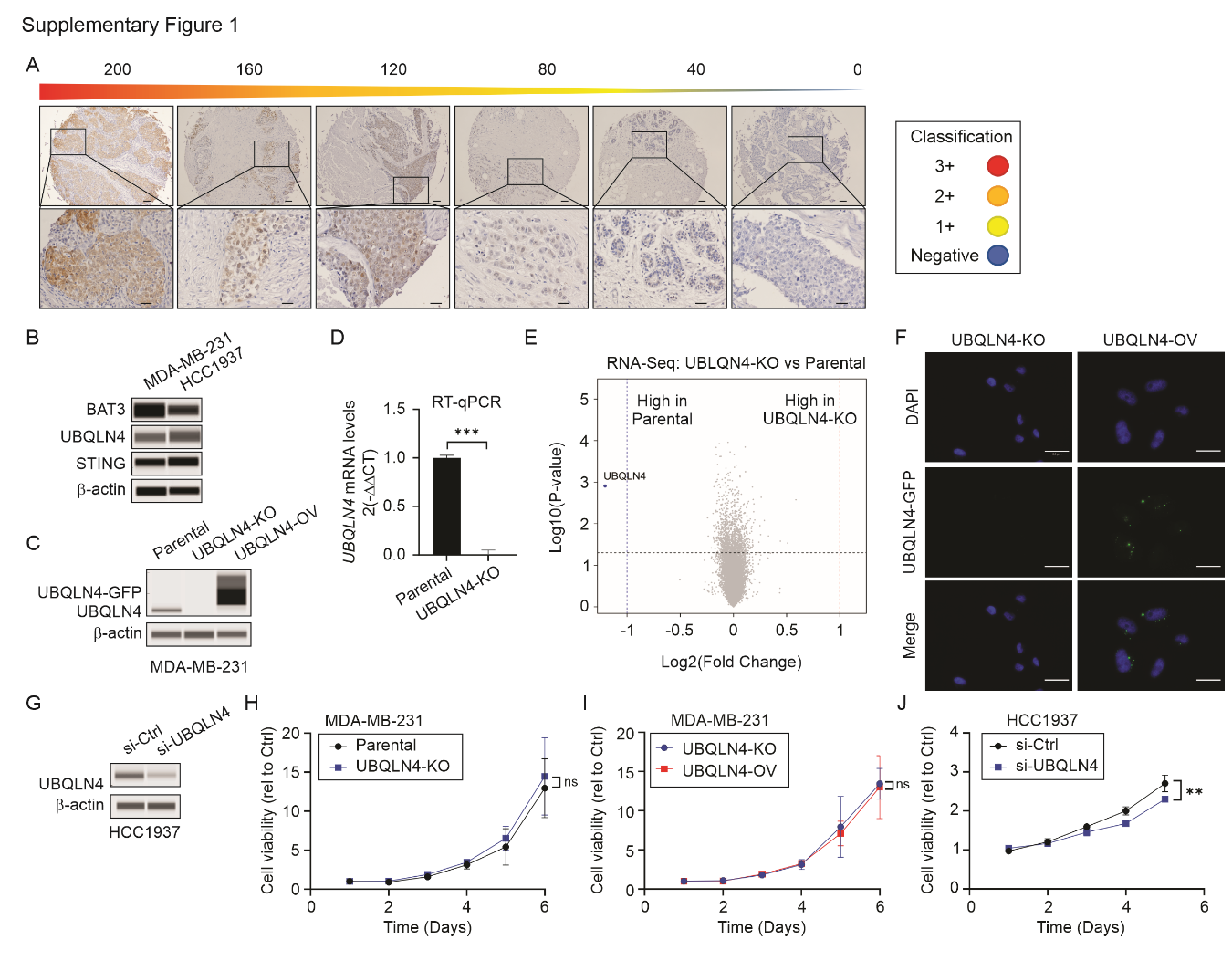
**

**Figure S1. UBQLN4 is upregulated in TNBC**

**A.** (Top) Representative images for each H-score in UBQLN4 IHC BC TMA. Cell detection/quantification images are shown below each IHC image. Scale bar=50µm. (Down) Magnification of the representative images. Scale bar=20 µm. Each tumor cell was classified according to 3,3’-diaminobenzidine (DAB) stain intensities, and the H-score values for each image were calculated by QuPath built-in “Positive cell detection”. **B.** Western blotting analysis for BAT3, UBQLN4, STING, and β-actin (loading control) in MDA-MB-231 and HCC1937 parental cell lines. **C.** Western blotting analysis for UBQLN4-GFP, UBQLN4, and β-actin (loading control) for MDA-MB-231 parental, *UBQLN4*-KO, and *UBQLN4*-OV cell lines. **D.** Comparison of *UBQLN4* mRNA levels between MDA-MB-231 parental and *UBQLN4*-KO cell lines using RT-qPCR (Mann-Whitney U test). **E.** Volcano plot showing the mRNA transcriptional changes between MDA-MB-231 parental and *UBQLN4*-KO cell lines. *UBQLN4* is indicated in blue. **F.** Immunofluorescence images for MDA-MB-231 *UBQLN4*-KO and *UBQLN4*-OV cell lines. Shown are UBQLN4 (GFP, green), Nucleus (DAPI, blue), and the merged images for each cell line. Scale bar=30µm. **G**. Western blotting analysis for UBQLN4 and β-actin (loading control) comparing HCC1937 cell line treated with si-Ctrl or si-UBQLN4. **H-I.** Cellular proliferation assay for MDA-MB-231 parental and *UBQLN4*-KO (**H**), MDA-MB-231 *UBQLN4*-KO, and *UBQLN4*-OV (**I**), and HCC1937 si-UBQLN4 and si-Ctrl (**J**) cell lines at indicated time points (Two-way ANOVA and Sidak’s multiple comparisons test). RT-qPCR, RNA-seq, and cellular proliferation assays for each cell line were performed in replicates (n=3).


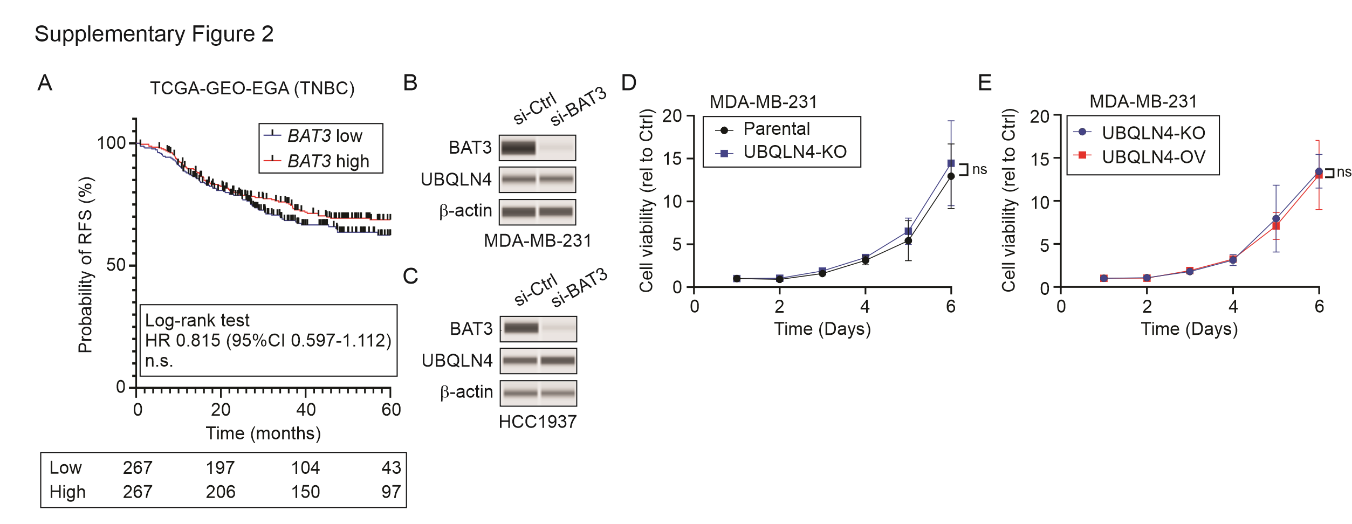


**Figure S2. BAT3 downregulation does not affect cell proliferation.**

**A.** RFS analysis for TNBC patients with low (n=267) versus high *BAT3* (n=267) mRNA expression in TCGA, GEO, and EGA databases combined. **B, C.** Western blotting analysis for BAT3, UBQLN4, and β-actin (loading control) comparing si-Ctrl and si-BAT3 in MDA-MB-231 (**B**) and HCC1937 (**C**) cell lines. **D, E.** Cellular proliferation assay for si-BAT3 and si-Ctrl in MDA-MB-231 (**D**) and HCC1937 (**E**) cell lines at indicated time points (Two-way ANOVA and Sidak’s multiple comparisons test).


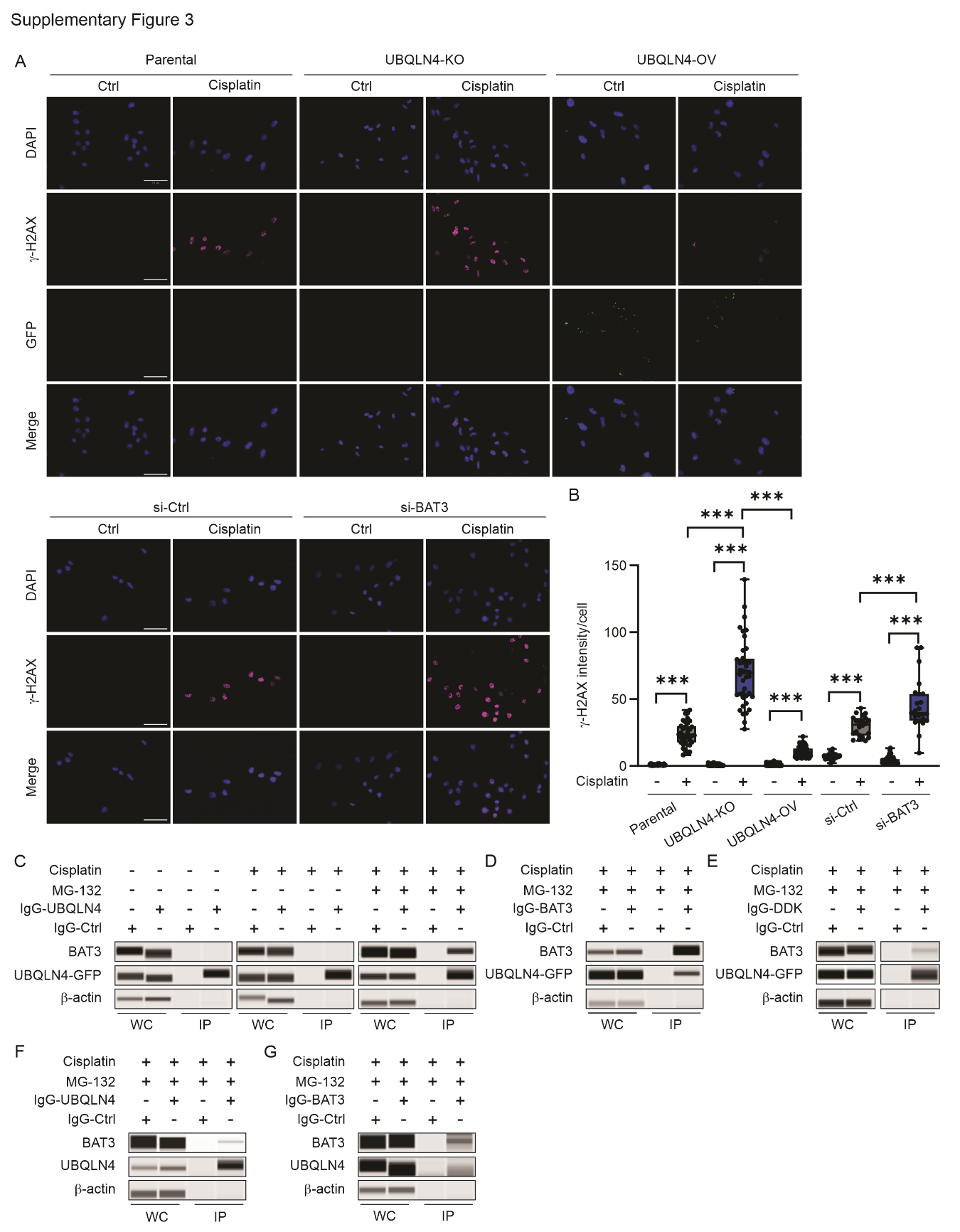


**Figure S3. UBQLN4 binds to BAT3 and ubiquitinated proteins during cisplatin-induced DNA damage**

**A**. Immunofluorescence staining for γ-H2AX performed in cisplatin-treated (5 μM, 8 hours) or non-treated MDA-MB-231 *UBQLN4*-KO, *UBQLN4*-OV, si-BAT3, and their respective control cell lines. Representative images are shown for γ-H2AX (Cy5, magenta), nucleus (DAPI, blue), UBQLN4 (GFP, green), and the merged images for each cell line/condition. Scale bar=70µm. **B.** Quantification of γ-H2AX fluorescence intensity (Two-way ANOVA and Sidak’s multiple comparisons test). **C.** Co-IP assay in MDA-MB-231 *UBQLN4*-OV cell lines untreated or treated with cisplatin (5 μM) ± MG-132 (5 μM) for 8 hours. Co-IPs were performed using UBQLN4 or control Ab. Protein levels were assessed in whole-cell lysates (WC) and co-IP fractions (IP). **D.** Co-IP assay in MDA-MB-231 *UBQLN4*-OV cell lines treated with cisplatin (5 μM) ± MG-132 (5 μM) for 8 hours. Co-IPs were performed using BAT3 or control Ab. Protein levels were assessed in whole-cell lysates (WC) and co-IP fractions (IP) **E.** Co-IP assay in MDA-MB-231 overexpressing *UBQLN4* and *UBB* that were treated with cisplatin (5 μM) + MG-132 (5 μM) for 8 hours. Co-IPs were performed using DDK tag or control Ab. Protein levels were assessed in whole-cell lysates (WC) and co-IP fractions (IP). **F, G.** Co-IP assay in MDA-MB-231 parental cell lines treated with cisplatin (5 μM) + MG-132 (5 μM) for 8 hours. Co-IPs were performed using UBQLN4 or control Ab (**F**), and BAT3 or control Ab (**G**). Protein levels were assessed in whole-cell lysates (WC) and co-IP fractions (IP). Cellular proliferation assays in each cell line were performed in replicates (n=3).


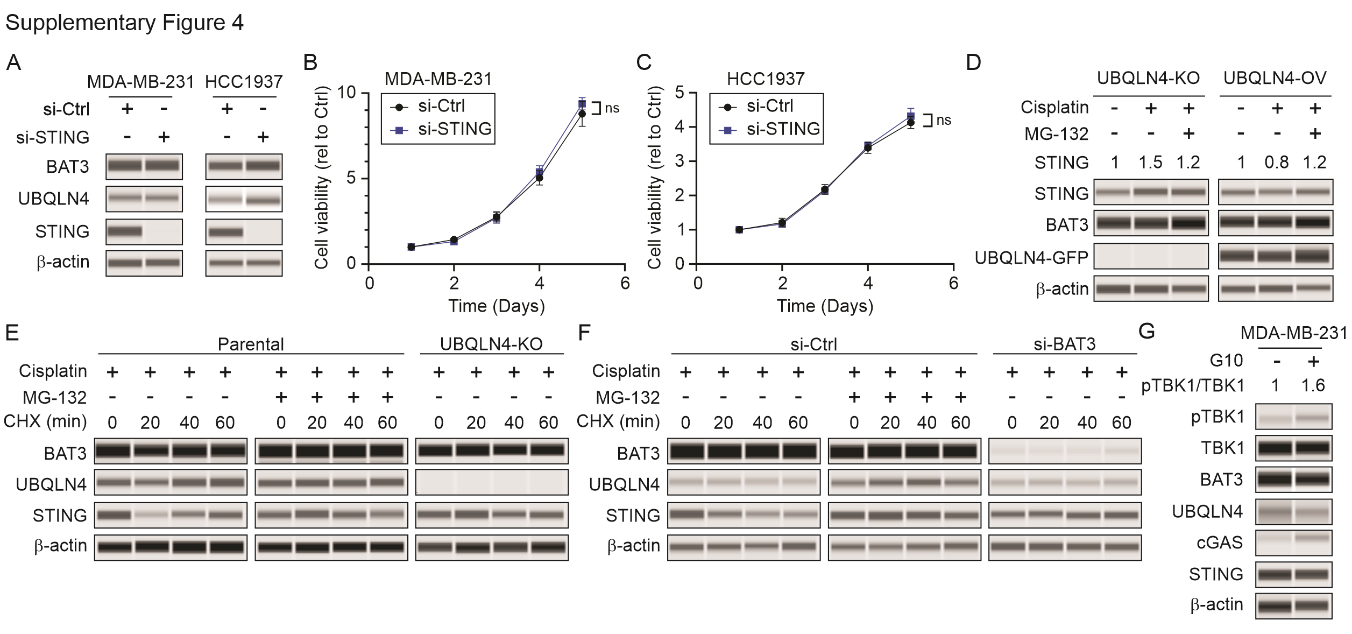


**Figure S4. UBQLN4 mediates STING proteasomal degradation**

**A.** Western blotting analysis for BAT3, UBQLN4, STING, and β-actin (loading control) comparing si-Ctrl and si-STING in MDA-MB-231 and HCC1937 cell lines. **B, C.** Cellular proliferation assay for si-STING and si-Ctrl in MDA-MB-231 (**B**) and HCC1937 (**C**) cell lines at indicated time points (Two-way ANOVA and Sidak’s multiple comparisons test). **D.** Western blotting analysis for STING, BAT3, UBQLN4-GFP, and β-actin (loading control) comparing MDA-MB-231 *UBQLN4*-KO and *UBQLN4*-OV cell lines. Cell lines were treated with cisplatin (5 μM) $\pm$ MG-132 (5 μM) for 8 hours. STING levels were quantified relative to respective controls. **E, F.** Western blotting images for CHX assay in MDA-MB-231 parental, *UBQLN4*-KO, si-BAT3, and si-Ctrl cell lines treated with cisplatin (5 μM) ± MG-132 (5 μM) for 8 hours. **G.** Western blotting analysis for STING pathway molecules (pTBK1, TKB1, BAT3, UBQLN4, cGAS, and STING) and β-actin (loading control) in MDA-MB-231 cell lines untreated or treated with G10 (25 μM, 8 hours). pTBK1/TBK1 ratio was quantified relative to respective controls.

**
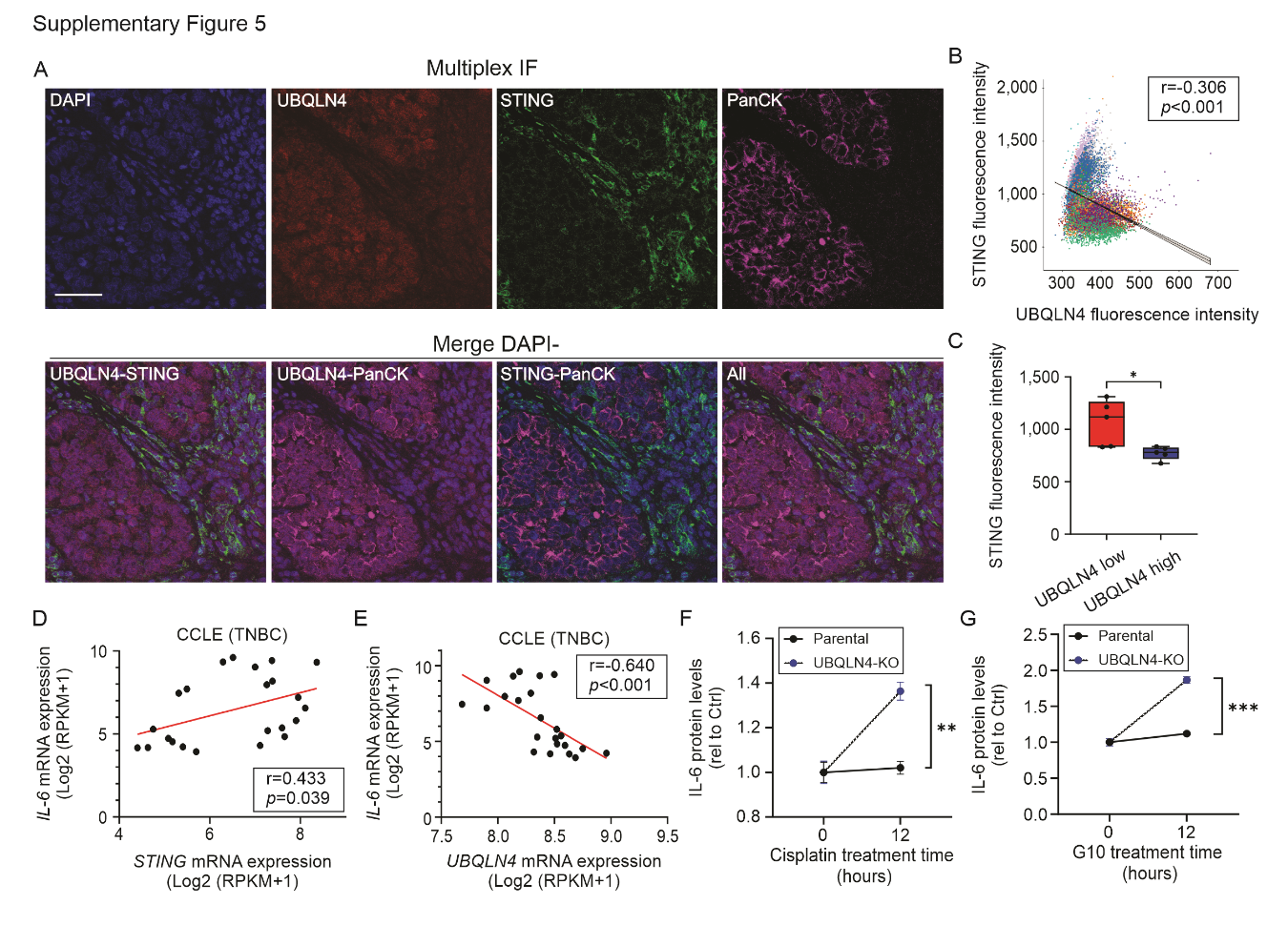
**

**Figure S5. UBQLN4 regulates STING levels and downstream activation**

**A.** Multiplex IF for UBQLN4, STING, and PanCK were performed in FFPE slides obtained from 10 primary TNBC tumors. Representative images are shown for UBQLN4 (AF594, red), STING (AF488, green), PanCK (AF647, magenta), and nucleus (DAPI, blue), and the merged images for each condition. Scale bar=50µm. **B.** Correlation between UBQLN4 and STING protein levels for 13,890 tumor cells across 10 primary TNBC tissues in the multiplex IF analysis. **C.** Comparison of STING protein levels for patients with low (n=5) and high (n=5) UBQLN4 protein levels in the multiplex IF analysis. Patients were divided into two groups by median UBQLN4 protein levels (Mann-Whitney U test). **D.** Correlation between *STING* and *IL-6* mRNA levels for 23 TNBC cell lines in the CCLE database. **E.** Correlation between *UBQLN4* and *IL-6* mRNA levels for 23 TNBC cell lines in the CCLE database. **F, G.** Comparison of the IL-6 protein levels in the cell culture supernatants for MDA-MB-231 parental and *UBQLN4*-KO cell lines during treatment. Cells were treated with cisplatin (5 μM, **F**) or G10 (25 μM, **G**) for 12 hours, and IL-6 protein levels in the cell culture supernatants were measured by ELISA. IL-6 levels were quantified relative to control. Cellular proliferation and ELISA assays in each cell line were performed in replicates (n=3).

**
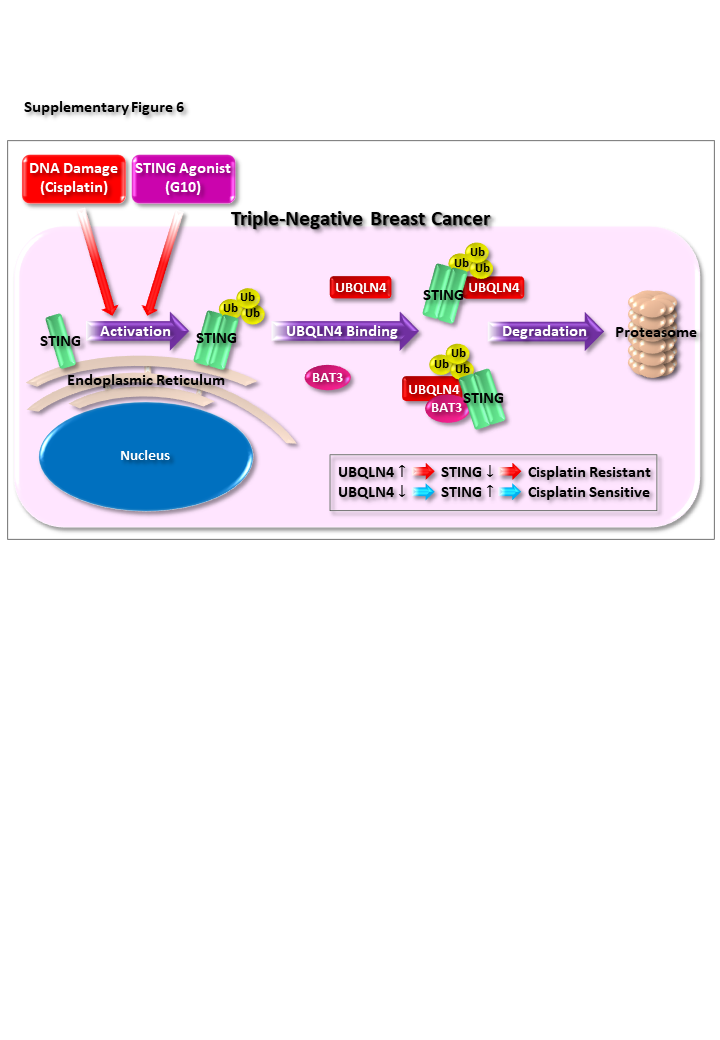
Figure S6. Graphical abstract summarizing the findings of the current study.**

**
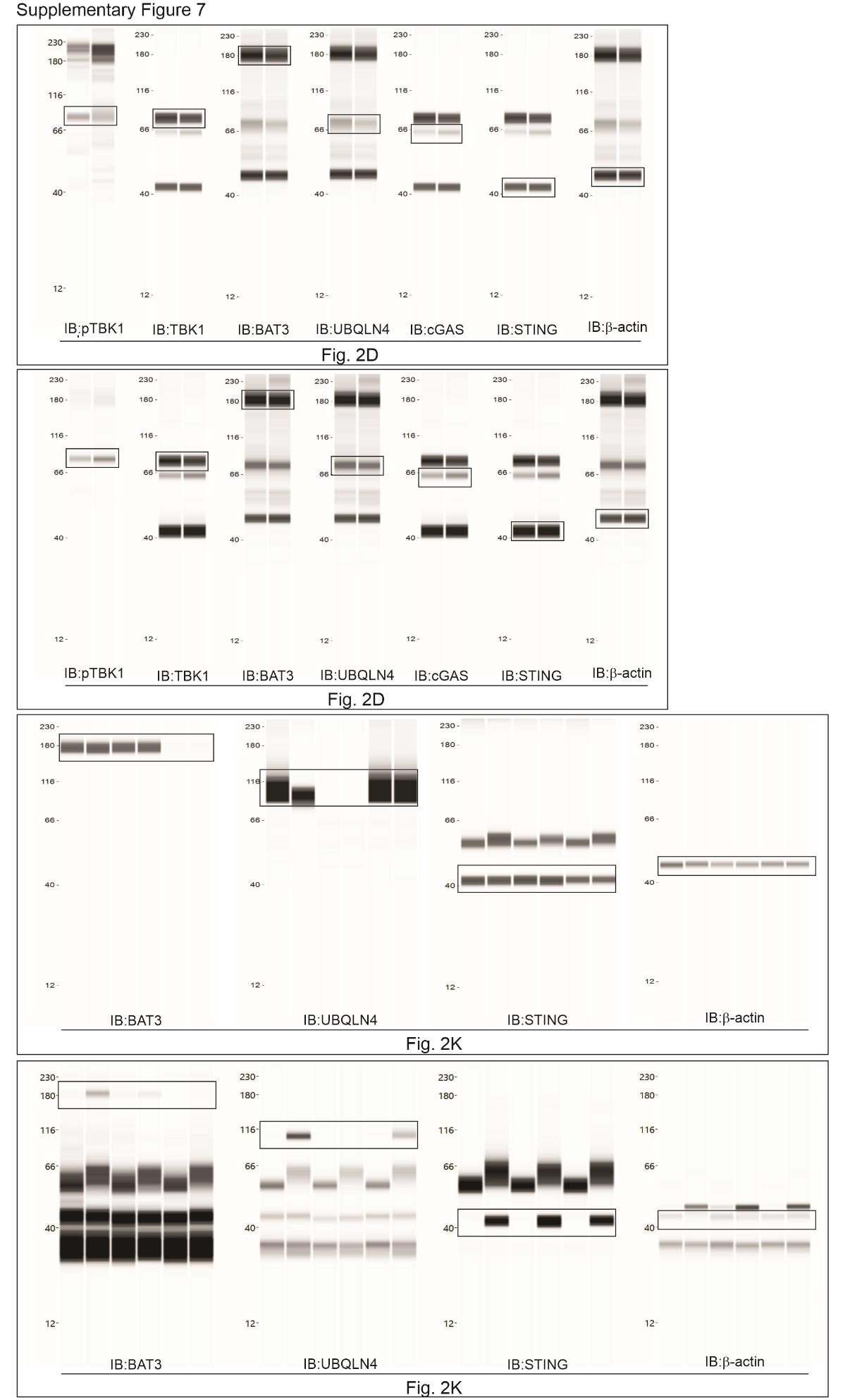
**

**Figure S7. Uncropped western blotting images**

Uncropped western blotting images for **Figure 2D** and 2**K** are shown.


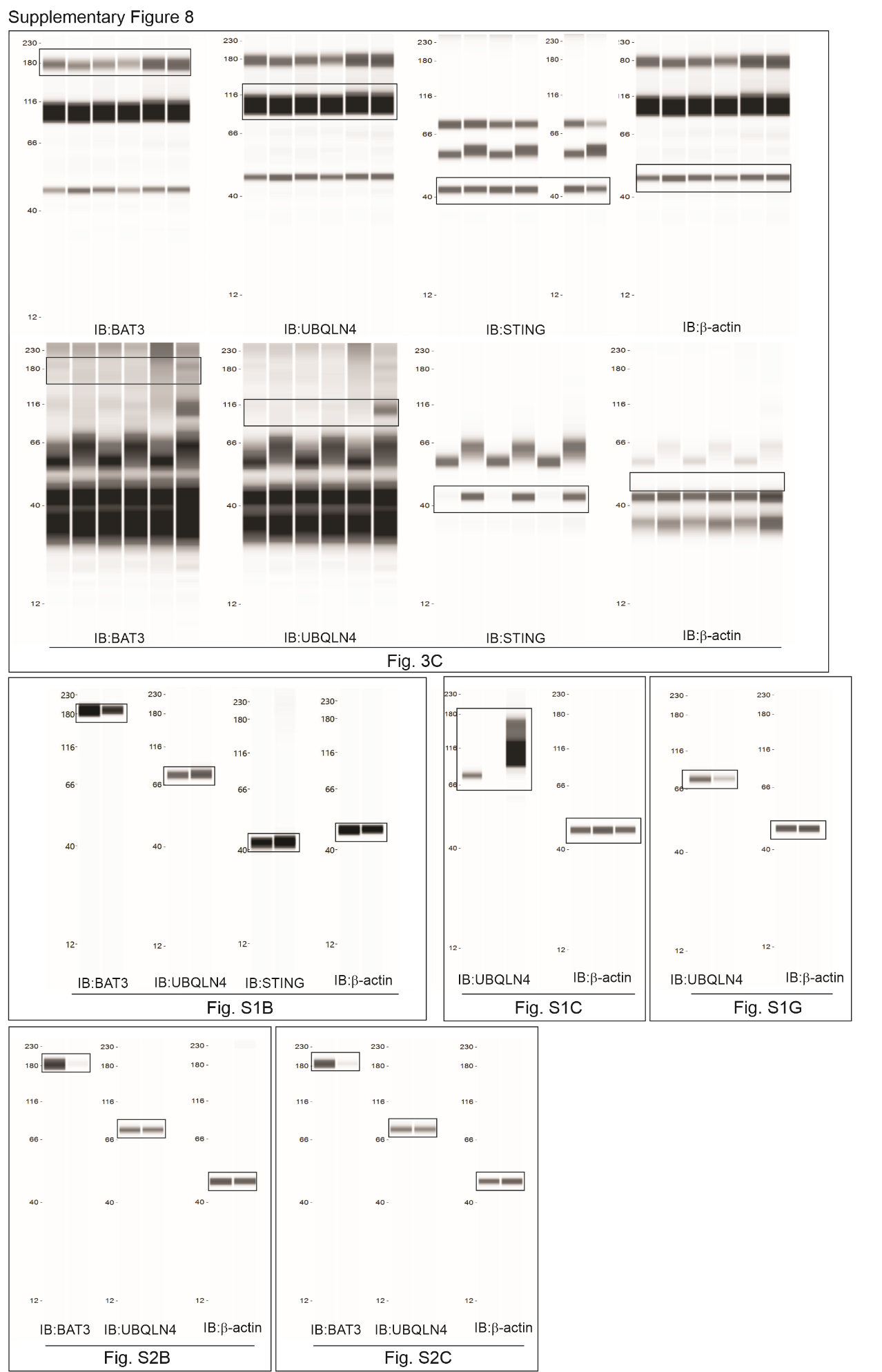


**Figure S8. Uncropped western blotting images**

Uncropped western blotting images for **Figure 3C, S1B, S1C, S1G, S2B,** and **S2C** are shown.

**
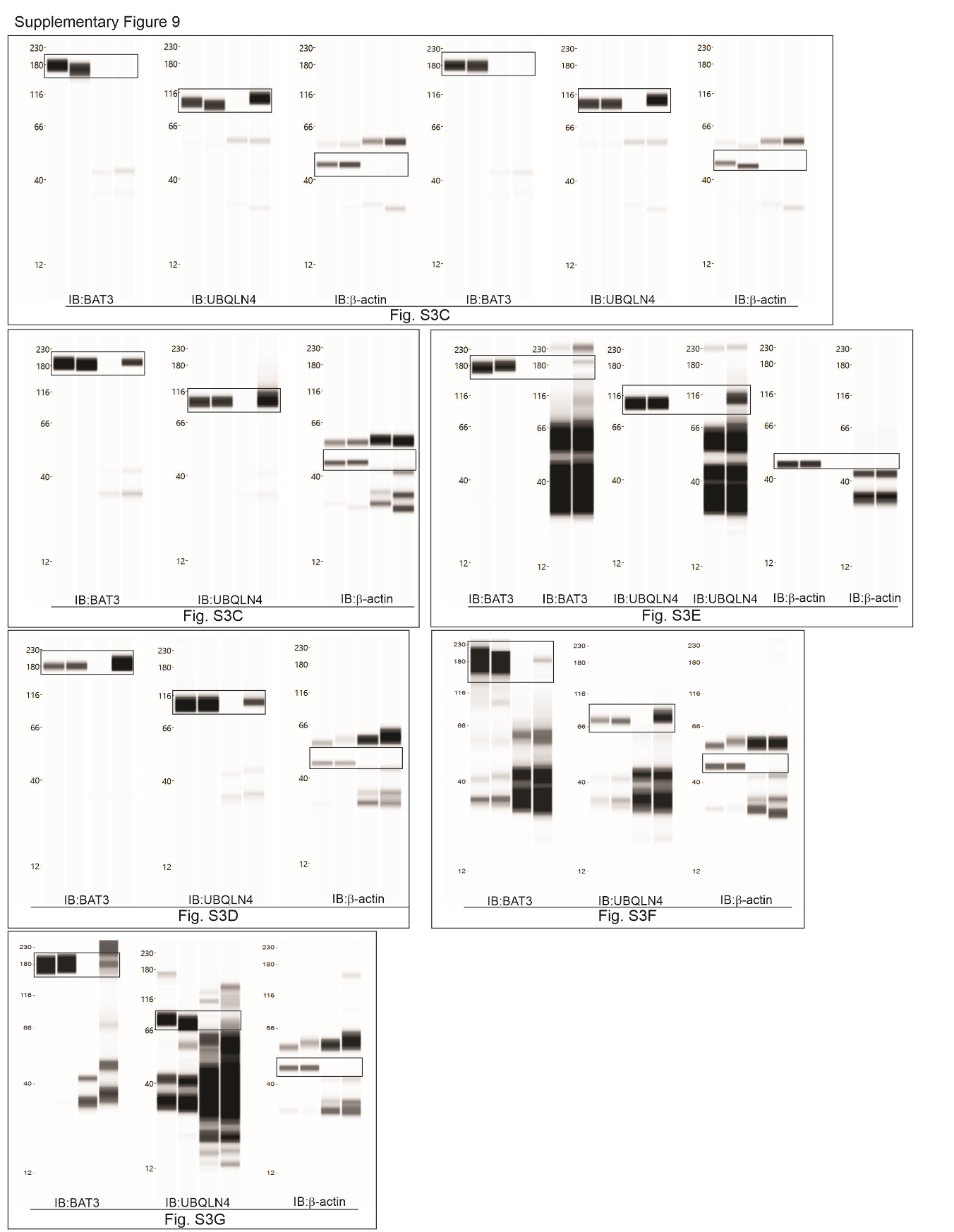
**

**Figure S9. Uncropped western blotting images**

Uncropped western blotting images for **Figure S3C, S3D, S3E, S3F,** and **S3G** are shown.

**
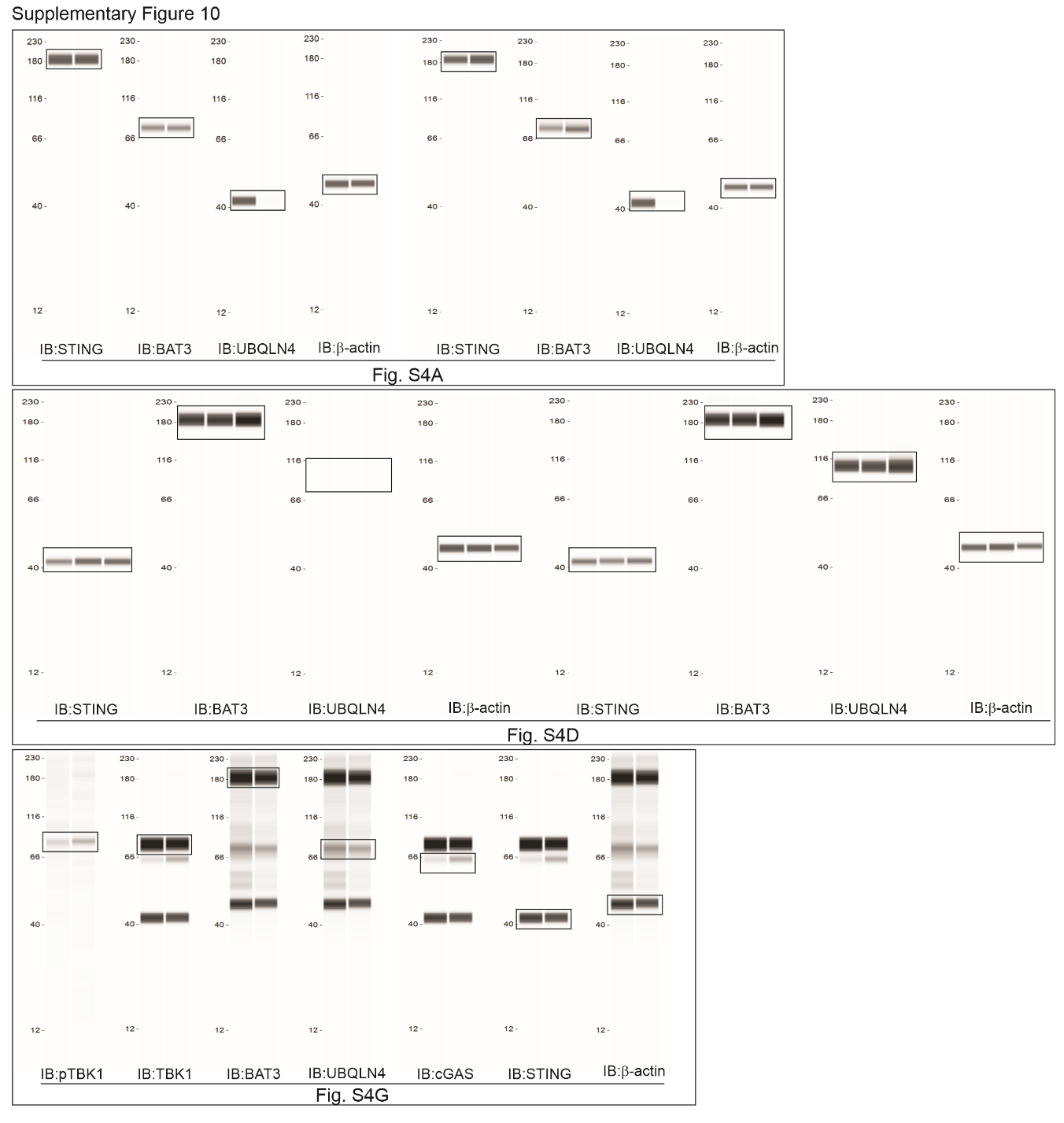
**

**Figure S10. Uncropped western blotting images**

Uncropped western blotting images for **Figure S4A, S4D,** and **S4G** are shown.

**
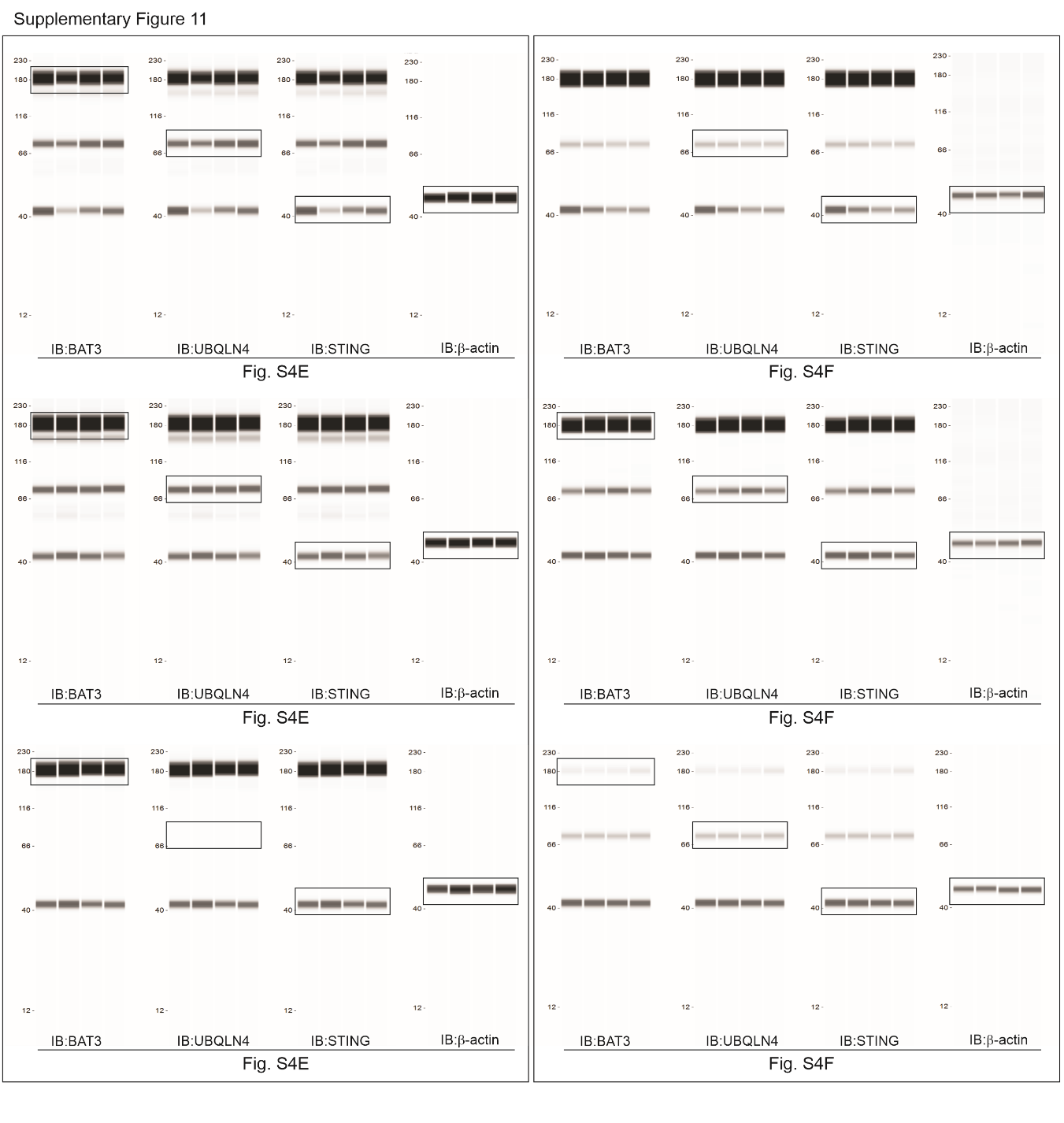
**

**Figure S11. Uncropped western blotting images**

Uncropped western blotting images for **Figure S4E** and **S4F** are shown.

**References**

1. Krupke DM, Begley DA, Sundberg JP, Bult CJ, Eppig JT. The Mouse Tumor Biology Database. *Nature Reviews Cancer*. 2008/06/01 2008;8(6):459-465. doi:10.1038/nrc2390

2. Gao H, Korn JM, Ferretti S, et al. High-throughput screening using patient-derived tumor xenografts to predict clinical trial drug response. *Nature Medicine*. 2015/11/01 2015;21(11):1318-1325. doi:10.1038/nm.3954

3. Bustos MA, Ono S, Marzese DM, et al. MiR-200a Regulates CDK4/6 Inhibitor Effect by Targeting CDK6 in Metastatic Melanoma. *J Invest Dermatol*. May 16 2017;doi:10.1016/j.jid.2017.03.039

4. Zhang X, Bustos MA, Gross R, et al. Interleukin enhancer-binding factor 2 promotes cell proliferation and DNA damage response in metastatic melanoma. *Clin Transl Med*. Oct 2021;11(10):e608. doi:10.1002/ctm2.608

5. Jachimowicz RD, Beleggia F, Isensee J, et al. UBQLN4 Represses Homologous Recombination and Is Overexpressed in Aggressive Tumors. *Cell*. Jan 24 2019;176(3):505-519.e22. doi:10.1016/j.cell.2018.11.024

6. Murakami T, Shoji Y, Nishi T, et al. Regulation of MRE11A by UBQLN4 leads to cisplatin resistance in patients with esophageal squamous cell carcinoma. *Mol Oncol*. Apr 2021;15(4):1069-1087. doi:10.1002/1878-0261.12929

7. Wang X, Bustos MA, Zhang X, et al. Downregulation of the Ubiquitin-E3 Ligase RNF123 Promotes Upregulation of the NF-κB1 Target SerpinE1 in Aggressive Glioblastoma Tumors. *Cancers (Basel)*. Apr 27 2020;12(5)doi:10.3390/cancers12051081

8. Bankhead P, Loughrey MB, Fernandez JA, et al. QuPath: Open source software for digital pathology image analysis. *Sci Rep*. Dec 4 2017;7(1):16878. doi:10.1038/s41598-017-17204-5

9. Schindelin J, Arganda-Carreras I, Frise E, et al. Fiji: an open-source platform for biological-image analysis. *Nature Methods*. 2012/07/01 2012;9(7):676-682. doi:10.1038/nmeth.2019

10. Bolte S, Cordelières FP. A guided tour into subcellular colocalization analysis in light microscopy. *J Microsc*. Dec 2006;224(Pt 3):213-32. doi:10.1111/j.1365-2818.2006.01706.x

11. Nguyen TTT, Shang E, Shu C, et al. Aurora kinase A inhibition reverses the Warburg effect and elicits unique metabolic vulnerabilities in glioblastoma. *Nature Communications*. 2021/09/01 2021;12(1):5203. doi:10.1038/s41467-021-25501-x

12. Koboldt DC, Fulton RS, McLellan MD, et al. Comprehensive molecular portraits of human breast tumours. *Nature*. 2012/10/01 2012;490(7418):61-70. doi:10.1038/nature11412

13. Carithers LJ, Ardlie K, Barcus M, et al. A Novel Approach to High-Quality Postmortem Tissue Procurement: The GTEx Project. *Biopreserv Biobank*. Oct 2015;13(5):311-9. doi:10.1089/bio.2015.0032

14. Goldman MJ, Craft B, Hastie M, et al. Visualizing and interpreting cancer genomics data via the Xena platform. *Nature Biotechnology*. 2020/06/01 2020;38(6):675-678. doi:10.1038/s41587-020-0546-8

15. Barretina J, Caponigro G, Stransky N, et al. The Cancer Cell Line Encyclopedia enables predictive modelling of anticancer drug sensitivity. *Nature*. 2012/03/01 2012;483(7391):603-607. doi:10.1038/nature11003

16. Yang W, Soares J, Greninger P, et al. Genomics of Drug Sensitivity in Cancer (GDSC): a resource for therapeutic biomarker discovery in cancer cells. *Nucleic Acids Research*. 2012;41(D1):D955-D961. doi:10.1093/nar/gks1111

17. Luna A, Elloumi F, Varma S, et al. CellMiner Cross-Database (CellMinerCDB) version 1.2: Exploration of patient-derived cancer cell line pharmacogenomics. *Nucleic Acids Research*. 2020;49(D1):D1083-D1093. doi:10.1093/nar/gkaa968

18. Győrffy B. Survival analysis across the entire transcriptome identifies biomarkers with the highest prognostic power in breast cancer. *Computational and Structural Biotechnology Journal*. 2021/01/01/ 2021;19:4101-4109. doi:<https://doi.org/10.1016/j.csbj.2021.07.014>
